# Beyond alcohol oxidase: the methylotrophic yeast *Komagataella phaffii* utilizes methanol also with its native alcohol dehydrogenase Adh2

**DOI:** 10.1093/femsyr/foab009

**Published:** 2021-02-18

**Authors:** Domen Zavec, Christina Troyer, Daniel Maresch, Friedrich Altmann, Stephan Hann, Brigitte Gasser, Diethard Mattanovich

**Affiliations:** Institute of Microbiology and Microbial Biotechnology, Department of Biotechnology, University of Natural Resources and Life Sciences (BOKU), Muthgasse 18, 1190 Vienna, Austria; CD-Laboratory for Growth-Decoupled Protein Production in Yeast, Department of Biotechnology, University of Natural Resources and Life Sciences, Muthgasse 18, 1190 Vienna, Austria; Institute of Analytical Chemistry, Department of Chemistry, University of Natural Resources and Life Sciences, Muthgasse 18, 1190 Vienna, Austria; Institute of Biochemistry, Department of Chemistry, University of Natural Resources and Life Sciences, Muthgasse 18, 1190 Vienna, Austria; Institute of Biochemistry, Department of Chemistry, University of Natural Resources and Life Sciences, Muthgasse 18, 1190 Vienna, Austria; Institute of Analytical Chemistry, Department of Chemistry, University of Natural Resources and Life Sciences, Muthgasse 18, 1190 Vienna, Austria; Institute of Microbiology and Microbial Biotechnology, Department of Biotechnology, University of Natural Resources and Life Sciences (BOKU), Muthgasse 18, 1190 Vienna, Austria; CD-Laboratory for Growth-Decoupled Protein Production in Yeast, Department of Biotechnology, University of Natural Resources and Life Sciences, Muthgasse 18, 1190 Vienna, Austria; Institute of Microbiology and Microbial Biotechnology, Department of Biotechnology, University of Natural Resources and Life Sciences (BOKU), Muthgasse 18, 1190 Vienna, Austria; CD-Laboratory for Growth-Decoupled Protein Production in Yeast, Department of Biotechnology, University of Natural Resources and Life Sciences, Muthgasse 18, 1190 Vienna, Austria

**Keywords:** methylotrophy, alcohol dehydrogenase, methanol, yeast, *Komagataella phaffii*, recombinant protein

## Abstract

Methylotrophic yeasts are considered to use alcohol oxidases to assimilate methanol, different to bacteria which employ alcohol dehydrogenases with better energy conservation. The yeast *Komagataella phaffii* carries two genes coding for alcohol oxidase, *AOX1* and *AOX2*. The deletion of the *AOX1* leads to the Mut^S^ phenotype and the deletion of *AOX1* and *AOX2* to the Mut^–^ phenotype. The Mut^–^ phenotype is commonly regarded as unable to utilize methanol. In contrast to the literature, we found that the Mut^–^ strain can consume methanol. This ability was based on the promiscuous activity of alcohol dehydrogenase Adh2, an enzyme ubiquitously found in yeast and normally responsible for ethanol consumption and production. Using ^13^C labeled methanol as substrate we could show that to the largest part methanol is dissimilated to CO_2_ and a small part is incorporated into metabolites, the biomass, and the secreted recombinant protein. Overexpression of the *ADH2* gene in *K. phaffii* Mut^–^ increased both the specific methanol uptake rate and recombinant protein production, even though the strain was still unable to grow. These findings imply that thermodynamic and kinetic constraints of the dehydrogenase reaction facilitated the evolution towards alcohol oxidase-based methanol metabolism in yeast.

## Teaser

To consume methanol, yeasts employ methanol oxidases, while bacteria use more energy efficient methanol dehydrogenases, and we show that dehydrogenase is a hidden layer of methanol consumption also in the yeast *Komagataella phaffii*.

## INTRODUCTION

Aerobic utilization of the C1 compound methanol is an exceptional capability of certain microbial species and it is a result of adaptation to specific niche environments where methanol is present. One such rather large environment is the Phyllosphere, the areal surface of plants and the associated decaying plant matter. The metabolism and decomposition of the cell wall constituent pectin serves as the source of methanol (Fall and Benson 1996; Galbally and Kirstine 2002; Kawaguchi *et al*. 2011; Vorholt 2012). These microorganisms, termed methylotrophs are phylogenetically very diverse and span across the domains. They comprise of Gram-positive and Gram-negative bacteria as well as yeasts. These microbes have developed different metabolic adaptations to achieve utilization of methanol and other C1 compounds as an energy and carbon source (Anthony 1982). The first step of methanol metabolism is the oxidation of methanol to formaldehyde, which represents a central key metabolite in most but not all of the known methylotrophic pathways. At this point the pathway partitions into the assimilation of formaldehyde into the biomass and dissimilation to CO_2_ for generating energy (Anthony 1982; Dijkhuizen, Levering and de Vries 1992; Yurimoto, Kato and Sakai 2005; Khadem *et al*. 2011). In yeasts these two pathways are also spatially separated into different compartments, the cytosol and peroxisome (van der Klei *et al*. 2006; Rußmayer *et al*. 2015).

The formation of formaldehyde from methanol is accomplished by different enzymes depending on the organism. Gram-negative methylotrophic bacteria have evolved a pyrroloquinoline quinone (PQQ) depended alcohol dehydrogenase (Adh) while their Gram-positive counterparts such as the thermophilic *Bacillus methanolicus* have an NAD^+^ dependent methanol dehydrogenase (Mdh) for the same purpose (Arfman *et al*. 1992; Dijkhuizen, Levering and de Vries 1992; Yurimoto, Kato and Sakai 2005; Krog *et al*. 2013). NAD^+^ dependent Mdhs have a low activity towards methanol (Krog *et al*. 2013; Ochsner *et al*. 2014) and the Gibbs free energy of methanol oxidation by Mdh is unfavorable at mesophilic temperatures compared to either Aox or the PQQ dependent Adh reaction (Whitaker *et al*. 2015), which may explain why the use of Mdhs evolved in thermophilic bacteria. Methylotrophic yeasts such as *Komagataella phaffii* (*Pichia pastoris*) or *Ogataea polymorpha* depend on an alcohol/methanol oxidase (Aox/Mox) to convert methanol to formaldehyde (Yurimoto, Kato and Sakai 2005; Yurimoto, Oku and Sakai 2011). This reaction implies two disadvantages: (i) Alcohol oxidase produces H_2_O_2_ which necessitates the localization of the enzyme to the peroxisome where the harmful H_2_O_2_ can be safely degraded without harming the cell (van der Klei *et al*. 2006). (ii) As the electrons are directly transferred from methanol to O_2_ they do not pass the electron transport chain, so that the ATP yield and subsequently the biomass yield (Y_X/S_) are reduced (Sheehan *et al*. 1988; Whitaker *et al*. 2015). A working hypothesis is that yeasts developed Aox as an alternative, thermodynamically feasible methanol oxidizing reaction with faster kinetics at lower methanol concentrations at the expense of energy efficiency, as they do not have PQQ enzymes available and evolved in mesophilic environments.

*Komagataella* *phaffii* contains two genes coding for alcohol oxidase. Based on the presence of intact *AOX* genes and the ability to utilize methanol, three methanol utilization (Mut) phenotypes of *K. phaffii* are defined. The wild type phenotype is Mut^+^, a ∆*aox1* strain is called Mut^S^ and a ∆*aox1*∆*aox2* strain is called Mut^–^ for methanol utilization positive, slow and negative, respectively. The Mut^–^ phenotype is generally regarded as incapable of utilizing methanol (Cregg *et al*. 1989; Sreekrishna *et al*. 1989; Chiruvolu, Cregg and Meagher 1997). However, in a recent study we found evidence that the Mut^–^ phenotype facilitates a low but significant rate of methanol oxidation although no growth on methanol was supported (Zavec, Gasser and Mattanovich 2020). Similarly, Singh and Narang (2020) suggested that there might be some residual Aox activity or an Aox-independent pathway in Mut^–^ strains that leads to formaldehyde formation. Therefore, we set out to investigate whether there is any additional, Aox independent pathway for methanol oxidation in *K. phaffii*. Mut^–^ strains have a residual specific methanol uptake rate (q_MeOH_) of about 4 mg g^–1^ h^–1^ which is about 2% of a wild type Mut^+^ and 10% of a Mut^S^ strain (Zavec, Gasser and Mattanovich 2020). We hypothesized that native Adhs may elicit a side reaction on methanol and are responsible for the low but significant methanol uptake in Mut^–^ strains.

## MATERIALS AND METHODS

### Generation of *ADH* deletion and overexpression strains

To generate the ∆*adh2*, ∆*adh900* and ∆*adh2*∆*adh900* strains the previously described *K. phaffii* strain CBS2612 ∆*aox1*∆*aox2* (Mut^–^) with or without human serum albumin (HSA) overexpression were used as parents (Zavec, Gasser and Mattanovich 2020). First the *adh2*∆*::loxP-hphMX-loxP* strain was created using a split marker cassette carrying a hygromycin resistance cassette already described (Nocon *et al*. 2014). For ∆*adh900* a new split marker cassette was designed carrying a geneticin resistance (Gasser *et al*. 2013). The three new strains are found in Table 1. The *K. phaffii* strains X-33 and X-33 Adh2KO were added as a comparison (Nocon *et al*. 2014). The deletion strains were selected on YPD with either 200 µg mL^–1^ hygromycin or 500 µg mL^–1^ gentamicin or a combination of both. The strains were verified by PCR amplification and sequencing of the PCR amplicons. The *ADH* overexpression strains were created by amplifying the *ADH2* and *ADH900* genes from CBS2612 Mut^–^ genomic DNA. The fragments were assembled by overlap extension PCR to obtain the coding sequence without *Bsa*I and *Bbs*I restriction sites needed for Golden Gate cloning (Prielhofer *et al*. 2017). The created plasmids BB3aZ_pGAP_ADH2_cycTT and BB3aZ_pGAP_ADH900_cycTT were linearized with *Asc*I (New England Biolabs) and transformed into the Mut^–^ strain, selected on YPD with 25 µg mL^–1^ Zeocin creating Adh2OE and Adh900OE strains. Transformation was done by electroporation (Gasser *et al*. 2013).

**Table 1.** Overview of the strains used in this study.

Strain name	Genotype	Source
Mut^–^	CBS2612 ∆*aox1*∆*aox2*	(Zavec, Gasser and Mattanovich 2020)
Mut^S^	CBS2612 ∆*aox1*	(Zavec, Gasser and Mattanovich 2020)
Adh2KO	CBS2612 ∆*aox1*∆*aox2 adh2*∆*::loxP-hphMX-loxP*	This study
Adh900KO	CBS2612 ∆*aox1*∆*aox2 adh900*∆*::loxP-kanMX-loxP*	This study
AdhKO	CBS2612 ∆*aox1*∆*aox2 adh2*∆*::loxP-hphMX-loxP adh900*∆*::loxP-kanMX-loxP*	This study
X-33	X-33 wt	
X-33 Adh2KO	X-33 P*_GAP_-hSOD-AOX1*tt *adh2*∆*::loxP-hphMX-loxP*	(Nocon *et al*. 2014)
Adh2OE	CBS2612 ∆*aox1*∆*aox2* P*_GAP_ADH2*	This study
Adh900OE	CBS2612 ∆*aox1*∆*aox2* P*_GAP_ADH900*	This study
P*_FLD1_*Adh2	CBS2612 ∆*aox1*∆*aox2* P*_FLD1_ADH*	This study
P*_AOX1_*Adh2	CBS2612 ∆*aox1*∆*aox2* P*_AOX1_ADH2*	This study
Mut^S^ P*_AOX1_*vHH	CBS2612 ∆*aox1* P*_AOX1_vHH-ScCYC1*tt*-bleMX*	This study
Mut^–^ P*_AOX1_*vHH P*_AOX1_*Adh2	CBS2612 ∆*aox1*∆*aox2* P*_AOX1_vHH-ScCYC1*tt*-bleMX-* P*_AOX1_ADH2-ScCYC1*tt-*KanMX*	This study
Mut^–^ P*_AOX1_*vHH P*_FLD1_*Adh2	CBS2612 ∆*aox1*∆*aox2* P*_AOX1_vHH-ScCYC1*tt*-bleMX-*P*_AOX1_ADH2-ScCYC1*tt*-KanMX*	This study

The Mut^–^ P*_AOX1_*vHH strain carrying a single copy of the vHH expression construct from our previous study was transformed with BB3aK_pAOX1_ADH2_cycTT and BB3aK_pFLD1_ADH2_cycTT (linearized with AscI) and selected on YPD 25 µg mL^–1^ Zeocin and 500 µg mL^–1^ gentamicin creating the strains Mut^–^ P*_AOX1_*vHH P*_AOX1_*Adh2 and Mut^–^ P*_AOX1_*vHH P*_FLD1_*Adh2. As a comparison, CBS2612 Mut^S^ was also transformed with the pPM2pZ30_pAOX1_αMFvHH_CycTT vector carrying a codon optimized variable region of a camelid antibody (vHH) fused to a *Saccharomyces cerevisiae* α-mating-type secretion signal sequence (Zavec, Gasser and Mattanovich 2020). Selection was done on YPD with 25 µg mL^–1^ Zeocin. Prior to bioreactor cultivation the strains were screened, an average producer was selected and a working cell bank for bioreactor cultivations was prepared as described previously (Zavec, Gasser and Mattanovich 2020).

### Cell free extracts for alcohol dehydrogenase assays

The alcohol dehydrogenase activity in cell free extracts was assayed by washing 2 mL of a liquid overnight culture on YPD at 25°C with 1 mL PBS and resuspending it in 500 µL cell lysis buffer with glass beads. The modified lysis buffer consisted of 20 mM HEPES, 420 mM NaCl, 1.5 mM MgCl_2_, 10% glycerol, 1 SIGMAFAST™ Protease Inhibitor Cocktail Tablet per 50 mL (Sigma-Aldrich GmbH) (Karaoglan, Karaoglan and Inan 2015). The cultures were lysed by bead beating (FastPrep-24, MP Biomedicals, Inc.) for 3 × 20 s at 6 m s^–1^ with 1-minute cooling on ice in-between steps. After the lysis step, the cultures were centrifuged, and the supernatant was transferred to a fresh microcentrifuge tube and centrifuged again at 13 200 rpm for 30 min at 4°C to remove any carried over cell debris. After the second centrifugation step the supernatant was stored at −20°C till use.

### Alcohol dehydrogenase activity assay

Prior to activity measurement, the protein concentration of the cell free extracts was measured by Pierce™ BCA Protein Assay (Thermo Scientific, Inc.) and adjusted to a common concentration for all samples. Then 20 µL cell free extracts were added to the reaction buffer and equilibrated for 10–15 minutes before addition of 1 M of ethanol as a substrate. The total end volume was 300 µL. The absorbance measurements of NADH at 340 nm were done in a 96 well plate using a microplate reader (Tecan Group Ltd.). The reaction buffer consisted of 100mM MOPS; 5mM MgSO_4_; 2mM NAD^+^ at pH 8.9 modified from Ochsner *et al*. (2014) and the activity was calculated in mU mg^–1^ as described elsewhere (Müller *et al*. 2015).

### Bioreactor cultivations

Bioreactor cultivation experiments were carried out in a DASGIP® Parallel Bioreactor System (Eppendorf AG). The cultivations consisted of (i) a batch phase, (ii) a feed phase and (iii) a methanol only phase where there was no other carbon source available except methanol. The batch medium used was BSM with 40 g L^–1^ glycerol as a carbon source followed by a 50% glucose feed (Mellitzer *et al*. 2014). Depending on the cultivation the pH was set to 5.0 or 5.5 by addition of 12.5% or 25% NH_4_OH and 10% phosphoric acid. The glucose feed rate was controlled gravimetrically by a custom balance controlled script. The glucose feed was run for 24 h at a feed rate of 2.9 g h^–1^ or 3.39 g h^–1^ depending on the cultivation to increase the biomass concentration before measuring the methanol metabolism associated parameters in the methanol feed phase. We applied a similar method for measuring the methanol uptake rate based on methanol pulses as already published (Dietzsch, Spadiut and Herwig 2011). Methanol was pulsed up to 1.5% (v/v) at the beginning of the glucose feed to induce and adapt the culture. After the glucose feed finished a second methanol pulse to 1.5% (v/v) was applied and the methanol concentration was measured by HPLC (Shimadzu, Corp.) at intervals to assess the q_MeOH_. Cell dry weight (CDW) was determined prior to the methanol shot as described before (Zavec, Gasser and Mattanovich 2020).

The cultivation of the vHH expressing strains was performed with strategy B and strategy D for the Mut^S^ comparison (Zavec, Gasser and Mattanovich 2020). Strategy B is divided into three phases: (i) batch, (ii) methanol-glucose co-feed, (iii) methanol feed phase. In the batch phase 300 mL BSM with 40 g L^–1^ glycerol was used, followed by a 50% (w/w) glucose feed at 5.8 g L^–1^ for 25 h. A methanol pulse was applied at the start of the methanol-glucose co-feed phase. A 50% (v/v) methanol feed was started to keep the methanol concentration at a target of 1.0% to 1.5% (v/v) till the end of the cultivation. Strategy D consisted of four phases: (i) batch, (ii) glycerol feed phase, (iii) methanol-glucose co-feed and (iv) methanol feed phase. The growth limiting 100% methanol feed was increasing at a rate of f(x) = 0.028x + 0.6. Every cultivation was done in duplicates and the reported data is the average of the two repeats. Where indicated, OTR and heat of reaction were calculated as described in Zavec, Gasser and Mattanovich (2020).

### Quantification of the recombinant protein

Quantification of the secreted recombinant protein in the culture supernatant was done by the LabChip GX/GXII System (PerkinElmer) using the consumables Protein Express Lab Chip (760499, PerkinElmer) and Protein Express Reagent Kit (CLS960008, PerkinElmer) according to the supplier´s instructions.

### ^13^C-Methanol labeled bioreactor cultivation

The ^13^C-methanol labeling experiments were carried out in the bioreactor in a similar manner. The (i) batch phase and (ii) glucose feed phase were carried out as described earlier. The glucose feed rate was increased to 5.8 g L^–1^ to achieve a biomass concentration of approximately 100 g L^–1^ CDW. The pH was controlled at 5.5 by 12.5% NH_4_OH and 10% phosphoric acid. Two hours prior to the end of the glucose feed phase, the gassing was changed to synthetic air without CO_2_ (20% O_2_, 80% N_2_). At the end of the glucose feed phase 150 mL of the reactor volume was removed so that approximate 325 mL was left in each reactor. At this point a 50% methanol pulse with ^13^C isotope labeled methanol or ^12^C methanol as the unlabeled control was added and a subsequent feed was started to keep the methanol concentration between 1.0% and 1.5% (v/v). An HPLC sample was taken right after the pulse and later approximately every 24 h. Biomass samples were used to determine CDW and the ^13^C/^12^C biomass isotope ratio, and the supernatant was analyzed for the ^13^C content in the secreted recombinant proteins. A CO_2_ trap consisting of 1M NaOH was used to capture the reactor exhaust gas over a period of 24 h.

Metabolite sampling was done one hour after the methanol pulse and then approximately every 24 hours. Quenching was done as described before (Mattanovich *et al*. 2017). Briefly, the sampling port was flushed and immediately a 2 mL sample was taken and quenched in 8 mL quenching solution (60% methanol, 125 mM TRIS-HCl, 55 mM NaCl, pH 8.2; T −27°C). Then 500 µL of the quenched mixture was filtered through a 0.45 µm cellulose acetate filter (Sartorius Stedim Biotech GmbH) and washed with 10 mL quenching solution at −27°C. The filter was immediately stored at −70°C until metabolite extraction.

### Intracellular metabolite sample preparation and ^13^C labeling measurements

For the ^13^C/^12^C metabolite ratio analysis the quenched samples were extracted with boiling ethanol (Neubauer *et al*. 2012; Rußmayer *et al*. 2015). 4 mL of 75% ethanol at 85°C was added to quenched and frozen biomass samples. The samples were vortexed for 20 s and transferred to a water bath at 85°C for 3 minutes. The sample were vortexed again after 1.5 minutes and at the end of the incubation period for 10 s each followed by rapid cooling on dry ice, avoiding freezing of the ethanol solution. The cooled samples were centrifuged for 10 minutes at 4000 g and −20°C and decanted. The ethanolic supernatant was vacuum dried and stored until use at −70°C.

The ^13^C labeling patterns of free intracellular metabolites were analyzed via gas chromatography chemical ionization—time of flight mass spectrometry (GC-CI-TOFMS) according to Mairinger *et al*. and Chu *et al*. with minor modifications (Chu *et al*. 2015; Mairinger *et al*. 2015). Measurements were carried out with an Agilent 7890B gas chromatograph combined with an Agilent 7200B QTOFMS system showing a mass accuracy of < 5 ppm. Prior to analysis, a two-step derivatization based on ethoximation and subsequent silylation was performed online on a GERSTEL DualRail MultiPurposeSampler (MPS2, GERSTEL, Germany). Isotope interference correction for the contribution of heavy isotopes from the derivatization agents and the native metabolite itself was performed using the software Isotope correction toolbox (ICT) developed by Jungreuthmayer *et al*. (2015). M/z ratios and mass extraction windows of the fragments or adducts used for data evaluation were first chosen as described in Mairinger *et al*. (2015), but needed to be adapted for some metabolites due to matrix interferences or saturation effects (2-phosphoglycerate: 475.1583, ±50 ppm, 3-phosphoglycerate: 475.1583, -15/+50 ppm; citrate 481.1924, −15/+50 ppm; iso-citrate: 481.1924, ±50 ppm; threonine: 248.1133, ±50 ppm; valine: 290.1966, ±50 ppm; glycine: 292.1579, ±50 ppm).

### Protein identification and peptide profiling by liquid chromatography-electrospray ionization-mass spectrometry

The supernatants containing a total amount of 30 μg protein each were S-alkylated with iodoacetamide and further digested with Sequencing Grade Modified Trypsin (Promega Corp.). An aliquot of 5 μg of the peptide mixture was analyzed using a Dionex Ultimate 3000 system directly linked to a Q-TOF instrument (maXis 4G ETD, Bruker GmbH) equipped with the standard ESI source in the positive ion, data dependent acquisition mode, DDA mode ( = switching to MSMS mode for eluting peaks). MS scans were recorded (range: 150–2200 m/z, spectra rate: 1 Hz) and the six highest peaks were selected for fragmentation (CID mode). Instrument calibration was performed using ESI calibration mixture (Agilent Inc.). For separation of the peptides a Thermo BioBasic C18 separation column (5 μm particle size, 150 × 0.320 mm) was used. A gradient from 97% solvent A and 3% solvent B to 62.5% solvent B in 45 min was applied, followed by a 15 min gradient from 62.5% solvent B to 95% solvent B at a flow rate of 6 μL/min at 32°C. Solvent A: 65 mM ammonium formate buffer, pH 3.0; Solvent B: 80% Acetonitril (VWR LLC; BDH Prolabo) and 20% solvent A. DataAnalysis 4.0 (Bruker GmbH) was used for peptide evaluation.

For each of the analyzed peptides the ^13^C/^12^C ratio was calculated using line spectra intensities of one specific charge state after normalization according to the number of carbon atoms present (cysteine carbamidomethylation considered). Error values for the presence of other heavier isotopes such as for nitrogen, oxygen, hydrogen and sulfur were calculated from the theoretical isotopic patterns (IsotopePattern; Bruker GmbH) and used for the correction of the measured and normalized ratio of the monoisotopic mass to the other isotopomers of each of the peptides. For each sample, an average ^13^C % value was calculated considering all of the six analyzed peptides (Table S1, Supporting Information).

### Biomass and CO_2_ isotope ratio by elemental analysis isotope ratio mass spectrometry

The biomass samples were kept frozen at −70°C and were washed twice with PBS to remove any residual ^13^C methanol before analysis. The captured CO_2_ in the form of sodium carbonate/bicarbonate was precipitated with ethanol. About 200 mL of the NaOH capture solution was mixed with 800 mL of absolute ethanol and cooled on ice until the sodium carbonate/bicarbonate precipitated. The precipitate was filtered, and vacuum dried to remove any residual ethanol from the precipitation. The biomass and sodium carbonate/bicarbonate isotope ratios were determined with elemental analysis isotope ratio mass spectrometry (EA-IRMS) performed by Imprint analytics GmbH, Austria (Gassler *et al*. 2020).

### HPLC methanol measurements

Methanol concentrations were determined at line using HPLC (Shimadzu Corp.) with an Aminex HPX-87H (Bio-Rad Laboratories, Inc.) column. The mobile phase was 4 mM H_2_SO_4_ at 0.6 mL h^–1^ at 60°C. The RID-10A detector at 40°C was used (Shimadzu Corp.) (Pflügl *et al*. 2012).

## RESULTS

### Adh2 is the major ethanol dehydrogenase of *K. phaffii*

Alcohol dehydrogenases are notoriously promiscuous enzymes that generally show low specificity towards a specific alcohol (Verduyn *et al*. 1988; Sealy-Lewis and Fairhurst 1995; Krog *et al*. 2013). *Komagataella phaffii* encodes six alcohol dehydrogenases (Valli *et al*. 2016), among which *ADH2* and *ADH900* are the two most highly transcribed genes (Ata *et al*. 2018). Activity of cell free extracts against ethanol was used to confirm the successful deletion of the active alcohol dehydrogenases in our test strains, as the activity against methanol in cell free extracts was too low to directly measure the effect of *ADH2* and *ADH900* deletion. When *ADH2* was deleted, a substantial reduction of ethanol dehydrogenase activity by 93% was observed, further deletion of *ADH900* resulted in almost complete loss of activity (Table 2). The Mut^–^ AdhKO double deletion strain still had a residual activity of 8.0 mU mg^–1^ but this represented only 0.6% of the initial Mut^–^ activity. Thus, *ADH900* only represents a marginal activity compared to *ADH2*. Taken together, this confirms that irrespective of the Mut phenotype, *ADH2* is mainly responsible for the ethanol dehydrogenase activity.

**Table 2.** Alcohol dehydrogenase activity (mU mg^–1^) against ethanol in cell free extracts of *K. phaffii* with standard errors and sample size in parenthesis.

Strain	Genotype	Activity (mU mg^–1^)
Mut^–^	CBS2612 ∆*aox1*∆*aox2*	1293.8 ± 244.9 (3)
Mut^–^ Adh2KO	CBS2612 ∆*aox1*∆*aox2 adh2*∆*::loxP-hphMX-loxP*	80.8 ± 7.9 (6)
Mut^–^ AdhKO	CBS2612 ∆*aox1*∆*aox2 adh2*∆*::loxP-hphMX-loxP adh900*∆*::loxP-kanMX-loxP*	8.0 ± 0.4 (3)
X-33 (Mut^+^)	X-33 wild type	1196.5 ± 28.3 (7)
X-33 Adh2KO (Mut^+^)	X-33 P*_GAP_-hSOD-AOX1*tt *adh2*∆*::loxP-hphMX-loxP*	88.5 ± 2.1 (6)

### Native alcohol dehydrogenases cause methanol uptake in Mut^–^ strains

**Figure 1.**
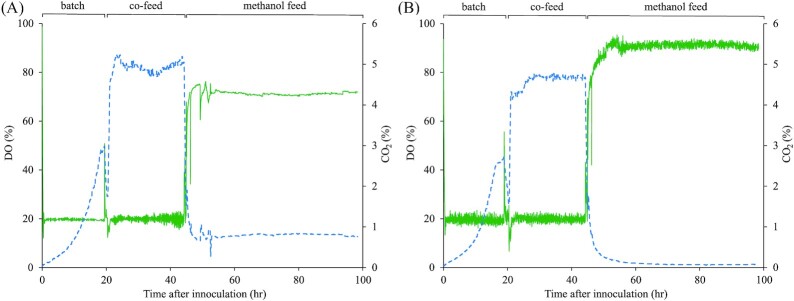
Dissolved oxygen and exhaust gas CO_2_ in bioreactor cultivations of **(A)**, Mut^–^ P*_AOX1_*HSA and **(B)**, Mut^–^ AdhKO. The full line represents the dissolved oxygen, the dotted line is CO_2_ concentration. Only one biological repeat of each strain is shown for clarity.

The Mut^–^ AdhKO strain (Mut^–^ ∆*adh2*∆*adh900)* that had nearly no Adh activity towards ethanol was tested for its specific methanol uptake (q_MeOH_) in the bioreactor. Additionally, the Mut^–^ Adh2KO (Mut^–^ ∆*adh2*) and Mut^–^ Adh900KO (Mut^–^ ∆*adh900*) single deletion strains and a strain overexpressing human serum albumin (Mut^–^ P*_AOX1_*HSA) in which we first observed methanol depletion (Zavec, Gasser and Mattanovich 2020) were used as comparison. Biomass was grown in glucose batch and fed batch cultures and induced with methanol during the limited glucose feed. Then a second methanol pulse of 1.5% (v/v) was applied and q_MeOH_ was measured by following the MeOH concentration by HPLC over time. Marked differences in dissolved oxygen and CO_2_ exhaust-gas concentrations were observed between the different strains indicating different degrees of methanol oxidation (Fig. 1, Table 3). By deletion of both *ADH2* and *ADH900* in the Mut^–^ background strain (AdhKO) q_MeOH_ was reduced to 0.7 mg g^–1^ h^–1^. According to the sterile bioreactor control published earlier (Zavec, Gasser and Mattanovich 2020) this value can be entirely explained by evaporation of methanol from the reactor medium by aeration and agitation. In contrast, the Mut^–^ P*_AOX1_*HSA had a measured q_MeOH_ of 5.1 mg g^–1^ h^–1^ which is consistent with previous observations (Zavec, Gasser and Mattanovich 2020). The single *ADH* deletion strains show that *ADH900* had no measurable effect on q_MeOH_, which is consistent with the ethanol dehydrogenase data where the *ADH900* deletion only had a marginal effect.

**Table 3.** Summary of the specific methanol uptake rates of the tested *K. phaffii* Mut^–^ strains. Average of two repeats is shown.

Strain name	Gene deletions	Adh overexpression	q_MeOH_ (mg g^–1^ h^–1^)
Mut^–^ P*_AOX1_*HSA	∆*aox1*∆*aox2*	none	5.1
Mut^–^ AdhKO	∆*aox1*∆*aox2 adh2*∆::HphR *adh900*∆::KanMX	none	0.7
Mut^–^ Adh900KO	∆*aox1*∆*aox2 adh900*∆::KanMX	none	5.5
Mut^–^ Adh2KO	∆*aox1*∆*aox2 adh2*∆::HphR	none	0.9
Mut^–^ Adh2OE	∆*aox1*∆*aox2*	P*_GAP_ADH2*	7.7
Mut^–^ Adh900OE	∆*aox1*∆*aox2*	P*_GAP_ADH900*	5.6
Mut^–^ P*_FLD1_*Adh2	∆*aox1*∆*aox2*	P*_FLD1_ADH2*	9.2
Mut^–^ P*_AOX1_*Adh2	∆*aox1*∆*aox2*	P*_AOX1_ADH2*	12.7

### Overexpression of *ADH2*, but not of *ADH900*, increases specific methanol uptake rate

Two strains overexpressing *ADH2* and *ADH900* with the constitutive glyceraldehyde 3-phosphate dehydrogenase (GAP) promoter (Adh2OE and Adh900OE) were generated and cultivated in the bioreactor as described above to determine the effect of the individual Adh overexpression on q_MeOH_. After the glucose feed phase the CDW reached an average concentration of 75.1 g L^–1^ (Adh2OE) and 74.9 g L^–1^ (Adh900OE). Then methanol was pulsed batch-wise to 10 g L^–1^ and q_MeOH_ was measured after 4.1 and 20.1 h (Table 4). After 4.1 h the methanol concentration was still in the range of 8g L^–1^ and thus not limiting. Therefore, we consider these data more reliable than q_MeOH_ calculated over 20.1 h where the methanol concentration was already in a sub saturation range below 0.4% (v/v). *ADH2* overexpression clearly shows an increase of q_MeOH_ over the wild type and of the *ADH900* overexpression strain. The *ADH900* overexpression strain has a q_MeOH_ which is approximately at the same level as in the Mut^–^ strain and the Adh900KO.

**Table 4.** Methanol concentrations and specific methanol uptake rates of ADH overexpressing *K. phaffii* Mut^–^ strains. Both biological repeats shown.

Strain name	*ADH* gene	CDW (g L^–1^)	MeOH at 0 h (g L^–1^)	MeOH at 4.1 h (g L^–1^)	q_MeOH_ at 4.1 h (mg g^–1^ h^–1^)	MeOH at 20.1 h (g L^–1^)	q_MeOH_ at 20.1 h (mg g^–1^ h^–1^)
Adh2OE	P_GAP_*ADH2*	72.9	10.3	8.0	7.65	1.2	6.18
Adh2OE	P_GAP_*ADH2*	70.7	10.7	8.4	7.80	1.3	6.51
Adh900OE	P_GAP_*ADH900*	71.3	10.3	8.6	5.91	2.8	5.23
Adh900OE	P_GAP_*ADH900*	71.7	10.3	8.7	5.30	2.9	5.09

### While most methanol is oxidized to CO_2_ in Mut^–^ strains, some is assimilated to primary metabolites and heterologous protein

To determine the fate of methanol in the *K. phaffii* Mut^–^ phenotype and to confirm the hypothesis that Adh2 and potentially Adh900 are responsible for methanol utilization, an experiment with ^13^C isotope labeled methanol was carried out. For this purpose, we used the already described Mut^–^ P*_AOX1_*HSA (Zavec, Gasser and Mattanovich 2020) as it provided the additional possibility to look at the labeling of a secreted recombinant protein. The second strain was Mut^–^ AdhKO, which was not capable of metabolizing methanol according to the previous experiments and served as a biological control. Each strain was cultivated in two repeats with ^13^C methanol and two parallels with ^12^C methanol as unlabeled control.

**Figure 2.**
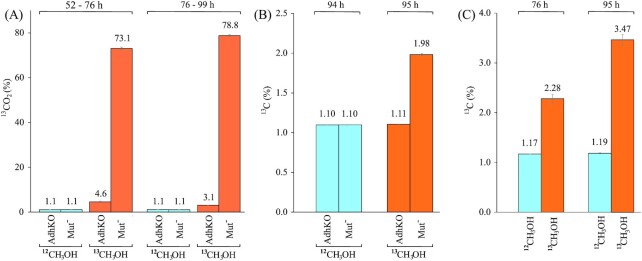
^13^C enrichment in **(A)**, the exhaust CO_2_, **(B)**, the biomass and **(C)**, the secreted recombinant protein. Error bars denote the standard deviation. ^13^CH_3_OH fed cultures are represented in orange, and ^12^CH_3_OH fed controls in blue. ^13^C enrichment is seen in all samples of the Mut^–^ strain while samples of the AdhKO strain do not enrich any ^13^C above the native content, as in the ^12^C control samples.

The CDW at the end of the glucose feed phase before the addition of methanol was similar for all 8 reactors at 99.3 ± 2 g L^–1^. After the glucose feed phase, either ^12^C or ^13^C methanol was added to the cultivation media, and kept at a concentration between 0.8% and 1.8% (v/v). In the methanol feed phase, the Mut^–^ AdhKO strain showed near zero CO_2_ in the exhaust gas while dissolved oxygen in the culture rose to nearly 100%, indicating that no substrate was oxidized in this phase. The Mut^–^ P*_AOX1_*HSA strain, in contrast maintained an exhaust CO_2_ level of 0.8% and a lower dissolved oxygen level (Fig. 1). The Mut^–^ P*_AOX1_*HSA strain led to lower DO and exhaust O_2_ concentrations and higher exhaust CO_2_ concentrations compared to the Mut^–^ AdhKO strain. Elemental analysis of the isotope ratios showed that the exhaust CO_2_ was highly enriched up to 79% with ^13^C isotope in the Mut^–^ P*_AOX1_*HSA strain with wild-type *ADH2* expression (Fig. 2A). In the Mut^–^ AdhKO, CO_2_ emission was substantially reduced and had lower ^13^C enrichment of 4.6%. The exhaust CO_2_ observed in the bioreactor cultivations of the Mut^–^ strain is therefore derived from oxidation of methanol by *K. phaffii*´s native Adh2. The methanol dissimilation ratio (q_CO2/_q_MeOH_) shows the relative amount of carbon flux through the dissimilatory pathway used for NADH and subsequently for ATP generation. Interestingly, the dissimilation ratio of the Mut^–^ P*_AOX1_*HSA strain is higher compared to the Mut^S^ strain, as determined in a later experiment (Table 5).

**Table 5.** Comparison of the specific methanol and oxygen uptake rates (q_O2_) and specific CO_2_ evolution rate (q_CO2_), respiratory quotient (RQ) and methanol dissimilation ratio. *^12^C isotope control of the labeling experiment.

	*Mut^–^AdhKO	*Mut^–^ P*_AOX1_*HSA	Mut^S^ P*_AOX1_*vHH	Mut^–^ P*_AOX1_*vHH P*_FLD1_*Adh2	Mut^–^ P*_AOX1_*vHH P*_AOX1_*Adh2
q_O2_ (mmol g^–1^ h^–1^)	0.032	0.183	1.152	0.444	0.541
q_CO2_ (mmol g^–1^ h^–1^)	0.016	0.118	0.684	0.259	0.338
RQ	0.48	0.64	0.59	0.58	0.62
q_MeOH_ (mg g^–1^ h^–1^)	1.30	4.52	37.06	7.85	11.12
q_MeOH_ (mmol g^–1^ h^–1^)	0.041	0.141	1.157	0.245	0.347
Methanol dissimilation ratio	78%	84%	59%	^†^106%	97%

* ^12^C isotope control of the labeling experiment.

† Values above 100% may be due to instrument imprecision or to additional metabolization of storage carbohydrates that show up as CO_2_ release.

The Mut^–^ P*_AOX1_*HSA biomass was slightly but significantly enriched with ^13^C compared to the unlabeled control (Fig. 2B). The Mut^–^ AdhKO strain showed no enrichment at all. This shows that the Mut^–^ phenotype is still capable of assimilating methanol although it cannot grow due to the lack of an alcohol oxidase, while the Mut^–^ AdhKO strains lose this ability completely. This was further confirmed by the increasing enrichment of ^13^C in the secreted protein which reached up to 3.5% at the end of the cultivation (Fig. 2C; Table S1, Supporting Information).

Over the course of the cultivation, three samples for metabolite analysis were taken at 1, 25, 52 h after the first methanol addition. The ^13^C enrichment in metabolite pools increased consecutively and was highest at the last sampling point. In particular, some key metabolites of the assimilatory methanol metabolism were highly ^13^C labeled as for instance sedoheptulose-7P (25%), fructose-6P (25%) and ribose-5P (11%). The lower glycolysis metabolites 3-phosphoglycerate (20%), 2-phosphoglycerate (19%) and the TCA cycle metabolite malate (16%) were also highly labeled (Table 6). As the lower glycolysis and TCA cycle are the source of amino acid precursors, this explains the ^13^C enrichment of the secreted recombinant protein.

**Table 6.** ^13^C metabolite labeling degree in % at different sampling time points. Values in bold are enriched above 10%.

	Time	1 h		25 h		52 h	
		Mut^–^ AdhKO	Mut^–^P*_AOX1_*HSA	Mut^–^AdhKO	Mut-P*_AOX1_*HSA	Mut^–^AdhKO	Mut^–^P*_AOX1_*HSA
Glycolysis	2-Phosphoglycerate	1.0%	3.5%	0.9%	**15%**	1.7%	**19%**
	3-Phosphoglycerate	1.8%	4.1%	1.1%	**16%**	1.1%	**20%**
TCA	Citrate	1.9%	1.8%	1.8%	2.2%	1.9%	2.4%
	Iso-citrate	1.1%	1.6%	1.1%	4.0%	1.1%	5.9%
	Malate	2.7%	4.3%	2.0%	8.9%	2.2%	**16%**
PPP/Methanol metabolism	Glucose-6-P	0.8%	2.5%	0.8%	**15%**	0.4%	**24%**
	Fructose-6-P	1.2%	2.9%	NA	**15%**	NA	**25%**
	6-Phosphogluconate	2.7%	2.6%	2.7%	3.3%	2.0%	6.3%
	Ribose 5-P	2.5%	2.6%	2.5%	5.2%	2.2%	**11%**
	Sedoheptulose 7-P	1.6%	2.9%	0.0%	**16%**	0.0%	**25%**
	Mannose-6-P	1.6%	3.1%	NA	**14%**	NA	**22%**
Amino acids	Threonine	1.4%	2.4%	2.1%	2.8%	1.9%	4.4%
	Valine	1.1%	1.2%	1.1%	1.4%	1.1%	1.6%
	Phenylalanine	1.7%	1.7%	1.9%	1.6%	1.8%	2.1%
	Isoleucine	0.7%	0.7%	1.1%	1.2%	1.2%	2.9%
	Leucine	0.9%	0.7%	1.2%	0.5%	1.2%	0.9%
	Lysine	0.8%	1.2%	2.9%	0.9%	0.9%	1.5%
	Glycine	2.5%	2.7%	2.6%	**11%**	3.1%	**31%**

### Overexpression of *ADH2* increases recombinant protein production

As shown in our previous study, the Mut^–^ strain is able to produce recombinant proteins when cultivated on methanol alone. This was quite unexpected as it was assumed that Mut^–^ strains cannot utilize methanol alone, so that they are typically cultivated with a co-substrate. The data presented here make it obvious, however, that methanol oxidation by *K. phaffii*´s native Adh2 enzyme results in a low but steady energy generation. To test whether alcohol dehydrogenase really impacts protein production, we transformed a Mut^–^ strain producing a camelide antibody fragment (Mut^–^ P*_AOX1_*vHH) with two *ADH2* overexpression constructs. *ADH2* overexpression was under control of two methanol responsive promoters with different expression strength, namely the P*_AOX1_* and P*_FLD1_*. The resulting strains Mut^–^ P*_AOX1_*vHH P*_AOX1_ADH2* and Mut^–^ P*_AOX1_*vHH P*_FLD1_ADH2* were tested in a bioreactor cultivation with strategy B as previously described (Zavec, Gasser and Mattanovich 2020). In addition, a Mut^S^ strain expressing VHH was cultivated as reference.

**Figure 3.**
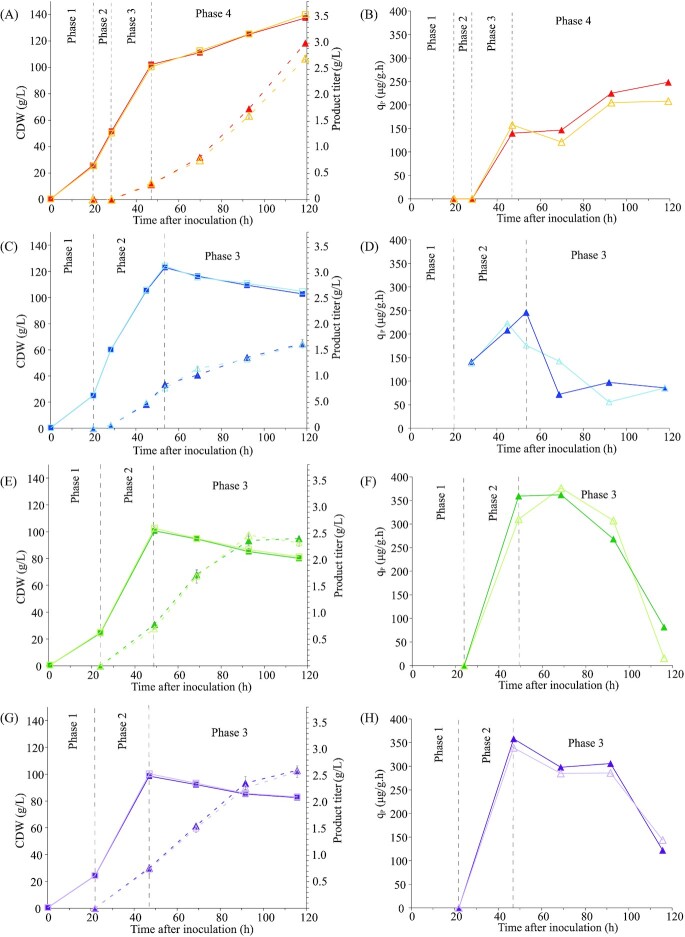
Biomass, recombinant protein titer and q_P_ profile for the Mut^S^ P*_AOX1_*vHH**(A, B)**, Mut^–^ P*_AOX1_*vHH from Zavec *et al*. (Zavec, Gasser and Mattanovich 2020) **(C,D)**, Mut^–^ P*_AOX1_*vHH P*_AOX1_*Adh2 **(E, F)** and Mut^–^ P*_AOX1_*vHH P*_FLD1_*Adh2 **(G, H)**. Full line with squares is the CDW. Dotted line with triangles is the product titer. Error bars indicate standard deviation of measurements. Closed dark and open light symbols represent the biological repeats, respectively.

As can be seen in fig. 3, overexpression of *ADH2* increases the productivity of the Mut^–^ production strain significantly. Compared to the parent strain Mut^–^ P*_AOX1_*vHH (Zavec, Gasser and Mattanovich 2020), the specific productivity (q_P_) in phase 3 is increased by 2.6 fold and approaches the q_P_ of the Mut^S^ strain. The Mut^S^ strain in phase 4 has an average q_P_ of 205 µg g^–1^ h^–1^. The *ADH2* overexpressing strains show a slightly higher q_P_ at 234 and 237 µg g^–1^ h^–1^ for the P*_AOX1_*Adh2 and P*_FLD1_*Adh2 overexpression, respectively. Notably, *ADH2* overexpression increased q_MeOH_ further, to a maximum of 12.7 mg g^–1^ h^–1^ (Table 3), and it increased methanol dissimilation even further compared to Mut^–^ P*_AOX1_*HSA towards the 100% mark (Table 5). In terms of productivity these strains are on par with the industry standard Mut^S^ strain for methanol induced recombinant protein production, however, at much lower oxygen demand and heat output (Table 7).

**Table 7.** Overview of the average specific productivity, oxygen transfer rates and heat evolution.

	q_P_ (µg g^–1^ h^–1^)	OTR (mM h^–1^)	Heat (W L^–1^)
Mut^S^ P*_AOX1_*vHH	205	137	17.5
*Mut^–^ P*_AOX1_*vHH	88	28	3.6
Mut^–^ P*_AOX1_*vHH P*_AOX1_*Adh2	234	49	6.2
Mut^–^ P*_AOX1_*vHH P*_FLD1_*Adh2	237	40	5.1

* Data from Zavec *et al*. (Zavec, Gasser and Mattanovich 2020).

## DISCUSSION

### *AOX* deficient *K. phaffii* can still utilize methanol

Methanol oxidation in methylotrophic yeasts is accomplished by alcohol oxidases (Aox) (Couderc and Baratti 1980; Cregg *et al*. 1989). Our previous study with *K. phaffii* lacking both *AOX* genes, however, revealed that a low level of methanol depletion of an unknown cause still occurred in these Mut^–^ strains even though there was no observable growth (Zavec, Gasser and Mattanovich 2020). One possible candidate for methanol oxidation in these strains are alcohol dehydrogenases, which are promiscuous enzymes with rather low substrate specificity and are generally more active towards higher alcohols (Verduyn *et al*. 1988; Sealy-Lewis and Fairhurst 1995; Krog *et al*. 2013; Zhang *et al*. 2017). We used this feature to detect and determine the presence of active alcohol dehydrogenases in cell free extracts of Adh deleted *K. phaffii* strains. In accordance with the data published by Karaoğlan *et al*. (2020), deletion of both *ADH2* and *ADH900* completely abolished the dehydrogenase activity towards ethanol. Strikingly, when the strains lacking ethanol dehydrogenase activity were tested for q_MeOH_ in bioreactors, methanol depletion was abolished to a level where it cannot be discriminated from evaporation anymore. On the other hand, deletion of *ADH900* in the Mut^–^ or the Mut^–^ Δa*dh2* background strain did not show any additional reduction of q_MeOH_. The effect of Δ*adh2* and Δ*adh900* on methanol was reflecting the dehydrogenase activity measurements, where we observed that *ADH900* accounts for only 6% of the total activity towards ethanol although expression levels of both Adh genes are similar and high on methanol (Prielhofer *et al*. 2015). In the same sense *ADH900* overexpression did not produce any distinct increase in q_MeOH_, suggesting that Adh900 is indeed much less active towards methanol (and ethanol) than Adh2.

The observed effects of both deletion and overexpression of *ADH2* make it obvious that this is the gene responsible for the observed methanol depletion. Thus *K. phaffii* can in fact utilize methanol by oxidation with the Adh2 enzyme. q_MeOH_ of the *ADH2* overexpression strains responds to the strength of the promoter used. From P*_GAP_*, to P*_FLD_* and P*_AOX1_* the q_MeOH_ increased by 1.5-, 1.8, and 2.5-fold compared to the control (Table 3). This represents 5% of the methanol uptake rate reported for Mut^+^ and 30% of that of Mut^S^ strains. The reported values range as high as 240–250 mg g^–1^ h^–1^ for Mut^+^ (Barrigon, Valero and Montesinos 2015; Tomàs-Gamisans, Ferrer and Albiol 2018) and up to 62 mg g^–1^ h^–1^ for Mut^S^ strains (Dietzsch, Spadiut and Herwig 2011). So even though Adh2 was overexpressed with the same promoter as Aox1 this leads only to a minor increase of q_MeOH_ compared to the wild type and the Mut^S^. In terms of q_MeOH_ Adh2 cannot substitute quantitatively for Aox1 and Aox2.

### Alcohol dehydrogenase may have had an auxiliary role in the evolution of yeast methylotrophy

Methanol metabolism is divided into the assimilatory and the dissimilatory pathways. In *K. phaffii* the assimilatory pathway is localized in the peroxisome (Rußmayer *et al*. 2015) and the dissimilatory in the cytosol (Yurimoto, Kato and Sakai 2005; van der Klei *et al*. 2006; Vanz *et al*. 2012; Rußmayer *et al*. 2015). The ^13^C labeling experiments confirmed that the CO_2_ formation observed with the Mut^–^ strains is indeed sourced from methanol. Furthermore, metabolite labeling reveals that at least a small amount of carbon is assimilated into the biomass and can be found both in the recombinant protein and the biomass.

Hypothetically, using an alcohol dehydrogenase instead of an oxidase could enable yeasts to utilize methanol in a more efficient way. The additional NADH yield from methanol oxidation by an Adh would increase the ATP yield per methanol unit (Sheehan *et al*. 1988) and would therefore decrease the needed flux through the dissimilatory pathway. This would change the balance between the assimilatory and dissimilatory flux toward assimilation and increase the biomass yield (Y_X/S_). Why did the pathway evolve in such a suboptimal way that came with three disadvantages, despite alcohol dehydrogenases being ubiquitous in yeasts? It was energetically unfavorable, produced H_2_O_2_ as a byproduct and necessitated the localization of the pathway to the peroxisomes by the duplication of numerous genes from the pentose phosphate pathway. The answer might be in the notoriously low activity of Adhs towards methanol (Krog *et al*. 2013; Ochsner *et al*. 2014) and the unfavorable Gibbs free energy of the Adh reaction at mesophilic temperatures compared to either Aox or the PQQ dependent Adh reaction (Whitaker *et al*. 2015). Additionally, microbes seem to be selected towards faster growth rates rather than Y_X/S_ so that an increased rate of the reaction even at low methanol concentrations may have an advantage over optimal ATP generation (Anthony 1982). Evidence from the bacterial domain suggest that temperature might be the decisive factor here. Bacteria lack peroxisomes to shield them from the harmful H_2_O_2_, so evolution of an intracellular alcohol oxidase was restricted. Instead, two Adhs evolved in bacterial methylotrophs, the PQQ dependent Adh and the NAD^+^ dependent Adh. Reduced PQQ yields less ATP upon oxidation than NADH and therefore the PQQ dependent Adh is less efficient than the NAD^+^ dependent counterpart. However, the Gibbs free energy change upon PQQ reduction is larger (Anthony 1982; Sheehan *et al*. 1988; Whitaker *et al*. 2015). So far, only thermophilic Gram-positive methylotrophs have been found to use NAD^+^ dependent Mdh (Nazina *et al*. 1988; Arfman *et al*. 1989; Krog *et al*. 2013; Visser *et al*. 2013). This might be due to thermodynamic constraints of this strategy at low temperatures and methanol concentrations, and improved kinetics at higher temperatures. The mesophilic lifestyle of yeast might have directed evolution towards an Aox based reaction. This perspective suggests that the development of mesophilic synthetic methylotrophs based on methanol dehydrogenases is inherently problematic, which is reflected by the difficulty to engineer a strain capable of growth on methanol as a single carbon source (Müller *et al*. 2015; Whitaker *et al*. 2017; Bennett et al. 2018, 2020; Meyer *et al*. 2018; Chen *et al*. 2020). In our case even a natural methylotroph already adapted to methanol utilization was unable to grow with an Adh as the initial step of methanol oxidation. Very recently, Espinosa *et al*. (2020) observed minor growth of *S. cerevisiae* on methanol when co-fed with yeast extract, and referred this to its native Adh activity.

**Figure 4.**
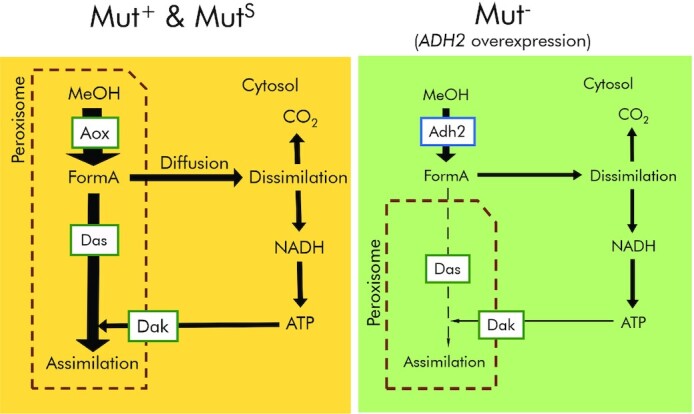
Simplified pathway comparing the localization of methanol utilization in the Mut^+^ and Mut^S^ strains to the Mut^–^ strain. Shown are methanol (MeOH), formaldehyde (FormA), the reduced cofactor nicotinamide adenine dinucleotide (NADH), adenosine triphosphate (ATP), and the enzymes alcohol oxidase (Aox), dihydroxyacetone synthase (Das) and dihydroxyacetone kinase (Dak).

The Adh2 present in *K. phaffii* is active towards methanol and in the Mut^–^ background strain it facilitates a q_MeOH_ that is beyond the reported non-growth associated maintenance energy (NGAME) of 2.81 mg g^–1^ h^–1^ (Tomàs-Gamisans, Ferrer and Albiol 2018). This could have served as the source for evolution towards methylotrophy, providing initially an additional energy source via methanol dissimilation. In the Mut^–^ strain more than 80% of methanol is oxidized to CO_2_ (a ratio that even increases to 100% when *ADH2* is overexpressed). In the presence of alcohol oxidase (Mut^+^ strain), only 50–80% of methanol is dissimilated to CO_2_ (Jordà et al. 2012, 2013; Vanz *et al*. 2012; Tomàs-Gamisans, Ferrer and Albiol 2018). According to the published model of yeast methanol metabolism, formaldehyde needs to diffuse into the cytosol to be dissimilated after being formed in the peroxisome. In some perspective the dissimilated formaldehyde is an overflow of the peroxisomal assimilation pathway (Douma *et al*. 1985; van der Klei *et al*. 2006). Our data suggest that the co-localization of methanol oxidation with the assimilatory pathway has been a key driver to evolve growth on methanol. While Adh2 is a cytosolic enzyme (Karaoglan, Karaoglan and Inan 2015; Valli *et al*. 2020) both Aox1 and Aox2 are peroxisomal matrix proteins. Contrary, in the Mut^–^ strain formaldehyde is formed in the cytosol where its oxidation is located which may explain the predominance of dissimilation in these strains (Fig. 4).

### *ADH2* overexpression is a useful tool to enhance recombinant protein production in Mut^–^strains

Although protein synthesis and production is an energy demanding process microbes evolved to prioritize it even under severe energy restrictions and starvation. This enables them to respond to changes in the environment by replacing enzymes and remodeling the metabolism to gain, for example, access to alternative energy and carbon sources (Jewett *et al*. 2009). This may explain why the Mut^–^ strain is capable of producing recombinant proteins while having a severely restricted q_MeOH_ and why the rather modest increase in q_MeOH_ by the *ADH2* overexpression has such a positive impact on both recombinant protein titers and productivity. Notably, even with *ADH2* overexpression, the Mut^–^ strains are still unable to grow on methanol alone.

*ADH2* overexpression increases productivity of the Mut^–^ strains to the level of the Mut^S^ strain, an industry standard, but the q_P_ profile is different. While q_P_ steadily increases for the Mut^S^ strain during the cultivation and peaks at the end, the Mut^–^*ADH2* overexpressing strains start at a much higher q_P_, than peak around the midpoint and decrease towards the end, forming a bell shape. Thus, using the Mut^–^*ADH2* overexpressing strains the final titer is already reached earlier in the cultivation, shortly after 92 h. In conclusion the Mut^–^*ADH2* overexpression strain can produce recombinant proteins at the same level as Mut^S^ while still retaining the benefits of low oxygen uptake and heat output.

## CONCLUSION

The combined evidence gathered here has made us rethink the long-lasting concept that the Mut^–^ strains of *K. phaffii* (and other methylotrophic yeasts) lacking *AOX1* and *AOX2* cannot oxidize methanol and that methanol loss is due to evaporation (Cregg *et al*. 1989; Looser *et al*. 2015). We showed that methanol metabolism in these strains is active, relying on the promiscuous activity of the Adh2 enzyme. Carbon from methanol gets incorporated into metabolites, biomass and recombinant protein. Overexpression of *ADH2* has a significant positive effect on q_MeOH_ compared to the Mut^–^ strain and q_MeOH_ is well above the reported NGAME for methanol, yet biomass growth cannot be observed. The wild type *K. phaffii* strains exhibit multiple times higher q_MeOH_, suggesting that evolution of a peroxisomal alcohol oxidase was necessary for yeast cells to compete for resources and achieve a competitive growth rate. Finally, we highlighted the potential application of *ADH2* overexpression for recombinant protein production in an industrial scenario.

## Supplementary Material

foab009_Supplemental_File
