# Supplementary material for: Beyond alcohol oxidase: the methylotrophic yeast Komagataella phaffii utilizes methanol also with its native alcohol dehydrogenase Adh2
Source: FEMS Yeast Res. 2021 Feb 18;21(2):foab009. doi: 10.1093/femsyr/foab009 (PMC7972947; doi:10.1093/femsyr/foab009)
Supplement: foab009_Supplemental_File [file foab009_supplemental_file.pdf]

Supplementary File to

Beyond alcohol oxidase: The methylotrophic yeast *Komagataella phaffii* utilizes methanol also with its native alcohol dehydrogenase Adh2

Domen Zavec, Christina Troyer, Daniel Maresch, Friedrich Altmann, Stephan Hann, Brigitte Gasser, Diethard Mattanovich

Table S1. Number of ^{13}C atoms found in the HSA peptides analyzed.

		Number of ^{13}C atoms in the analyzed peptide							
	Carbon source	^{12}C methanol				^{13}C methanol			
	Sample Time	76 h		95 h		76 h		95 h	
Peptide	C atoms	R1	R2	R1	R2	R1	R2	R1	R2
DLGEENFK	41	0.49	0.53	0.53	0.49	0.86	0.91	1.25	1.33
SLHTLFGDK	46	0.55	0.53	0.56	0.56	1.07	1.14	1.92	2.02
AEFAEVSK	39	0.46	0.46	0.47	0.46	0.90	0.96	1.53	1.54
SHCIAEVE NDEMPAD LPSLAADF VESK	163	1.86	1.87	1.95	1.93	4.43	4.65	6.88	7.15
VFDEFKPL VEEPQNLIK	96	1.12	1.11	1.08	1.10	1.57	1.64	1.97	2.10
KVPQVSTP TLVEVSR	72	0.85	0.84	0.85	0.85	1.32	1.40	1.95	2.06
Total	457	5.34	5.34	5.45	5.39	10.15	10.70	15.50	16.18
Percent		1.17%	1.17%	1.19%	1.18%	2.22%	2.34%	3.39%	3.54%
Average with SD		1.17% ±0.00		1.19% ± 0.01%		2.28% ±0.08%		3.47% ±0.11%	
